# Prevalence of Multidrug-Resistant and Extended-Spectrum Beta-Lactamase-Producing *Shigella* Species in Asia: A Systematic Review and Meta-Analysis

**DOI:** 10.3390/antibiotics11111653

**Published:** 2022-11-18

**Authors:** Mohd Zulkifli Salleh, Nik Mohd Noor Nik Zuraina, Khalid Hajissa, Mohamad Ikram Ilias, Kirnpal Kaur Banga Singh, Zakuan Zainy Deris

**Affiliations:** 1 Department of Medical Microbiology & Parasitology, School of Medical Sciences, Universiti Sains Malaysia Health Campus, Kubang Kerian 16150, Malaysia; 2 Department of Zoology, Faculty of Science and Technology, Omdurman Islamic University, P.O. Box 382, Omdurman 14415, Sudan; 3 Department of Pediatrics, School of Medical Sciences, Universiti Sains Malaysia Health Campus, Kubang Kerian 16150, Malaysia

**Keywords:** *Shigella sonnei*, *Shigella flexneri*, *Shigella boydii*, *Shigella dysenteriae*, multidrug resistance, extended-spectrum beta-lactamase, epidemiology, Asia, systematic review, meta-analysis

## Abstract

Shigellosis remains one of the leading causes of morbidity and mortality worldwide and is the second leading cause of diarrheal mortality among all age groups. However, the global emergence of antimicrobial-resistant *Shigella* strains, limiting the choice of effective drugs for shigellosis, has become the major challenge in the treatment of *Shigella* infections. The aim of this systematic review and meta-analysis was to provide an updated picture of the prevalence of antimicrobial-resistant *Shigella* species in Asia. A comprehensive and systematic search was performed on three electronic databases (PubMed, ScienceDirect and Scopus), in which 63 eligible studies published between 2010 and 2022 were identified. From our meta-analysis of proportions using a random-effects model, the overall prevalence of *Shigella* spp. in Asian patients was estimated to be 8.0% (95% CI: 5.5–10.5). The pooled prevalence rates of multidrug-resistant (MDR) and extended-spectrum beta-lactamase (ESBL)-producing *Shigella* strains were 68.7% (95% CI: 59.9–77.5) and 23.9% (95% CI: 12.9–34.8), respectively. Concerning recommended antimicrobial drugs for *Shigella*, the prevalence of resistance was highest for ciprofloxacin (29.8%) and azithromycin (29.2%), followed by ceftriaxone (23.8%), in spite of their importance as first- and second-line treatments for shigellosis. In contrast, resistance to carbapenems, such as ertapenem (0.0%), imipenem (0.1%) and meropenem (0.0%), was almost non-existent among the 49 tested antibiotics. The significantly high prevalence estimation suggests that the multidrug-resistant *Shigella* is a pressing threat to public health worthy of careful and justified interventions. Effective antibiotic treatment strategies, which may lead to better outcomes for the control and treatment of shigellosis in Asia, are essential.

## 1. Introduction

The *Shigella* species is one the most prevalent diarrheal pathogens in Asia, particularly in South Asia [1], and is responsible for shigellosis, an intestinal infection that is also known as bacillary dysentery. Among the four *Shigella* spp.—*Shigella dysenteriae* (12 serotypes), *Shigella boydii* (23 serotypes), *Shigella flexneri* (12 serotypes) and *Shigella sonnei* (1 serotype)—more than 90% of global shigellosis cases are caused by *S. flexneri* and *S. sonnei* [2]. The current global epidemiology studies demonstrate that *S. sonnei* is increasingly overtaking *S. flexneri* to become the predominant species in some parts of the world, including Asia [3,4,5]. Shigellosis is characterized by the invasion and disruption of the epithelium lining the terminal ileum, colon and rectum, resulting in acute watery diarrhea and dysentery, with frequent bloody stools, fever and abdominal cramps [6]. The global burden of shigellosis is estimated to be 125 million episodes annually, with more than 200,000 deaths among all age groups [7]. The World Health Organization (WHO) recommended ciprofloxacin as the first-line antimicrobial therapy for all shigellosis patients with bloody diarrhea, irrespective of age. Pivmecillinam and ceftriaxone were recommended as the second-line treatments in all age groups, while azithromycin was recommended as an alternative for treatment in adults [8]. However, overuse or misuse of antibiotics in treating shigellosis increases antibiotic resistance, further limiting therapeutic options for *Shigella* infections.

Antimicrobial-resistant *Shigella* strains have become a major global concern and a significant threat to public health. Nowadays, the reliance on ciprofloxacin for shigellosis treatment has markedly prompted the spread of fluoroquinolone-resistant *S. sonnei* worldwide, which most likely evolved from a single common progenitor in South Asia around 2007 [9,10]. This single clonal expansion event was associated with classical sequential mutations in the quinolone resistance determining region; specifically, in *gyrA* codon 83 or 87 and *parC* codon 80 [9]. Back in the 1980s, a high proportion of Southeast Asian *S. sonnei* and *S. flexneri* strains were found to exhibit resistance to sulfonamides, tetracyclines and streptomycin due to the acquisition of plasmids harboring the *sul2*, *tetra* and *str*AB genes [10]. A surveillance study later reported that the majority of *Shigella* spp. isolated from Asian countries in the early 2000s were resistant to ampicillin and co-trimoxazole, among which *S. flexneri* and *S. sonnei* were found to be highly associated with ampicillin and co-trimoxazole resistance, respectively [11]. Subsequently, in the mid-2000s to early 2010s, horizontal transfer of the pKSR100 plasmid encoding for resistance to azithromycin from *S. flexneri* into other *Shigella* spp. has intensified the emergence of novel variants. For instance, the recent dominance of dual ciprofloxacin and azithromycin resistance in *S. sonnei* was reported in the late 2010s [12]. 

Apart from the overwhelming number of particular drug resistance strains, the emergence of multidrug resistance (MDR) is a burden in the treatment of shigellosis, and nearly half of *Shigella* strains around the world have now become resistant to multiple antibiotics [13]. Most of the MDR *Shigella* strains confer resistance to third-generation cephalosporins, such as ceftriaxone and cefoperazone, azithromycin and fluoroquinolones, commonly via point mutations and horizontal transfer of plasmid-mediated resistance mechanisms [3,13]. Third-generation cephalosporin resistance has also been linked to the production of extended-spectrum beta-lactamases (ESBLs), which confer resistance to the CTX-M, TEM and SHV-lactamase families [4]. The rising prevalence of ESBL-producing *Shigella* spp. in Asian countries [14,15] signals a wake-up call, requiring new approaches and alternative therapies in the management of shigellosis. Hence, updates on the antimicrobial resistance in *Shigella* spp. are necessary for effective and justified treatments to reduce the morbidity and mortality rates associated with shigellosis. The aim of this systematic review and meta-analysis (SRMA) was to provide a current and comprehensive pooled prevalence estimation of MDR- and ESBL-producing *Shigella* spp. in Asia from more than a decade of published empirical data.

## 2. Results

### 2.1. Study Selection and Characteristics of the Included Studies

**Figure 1:**
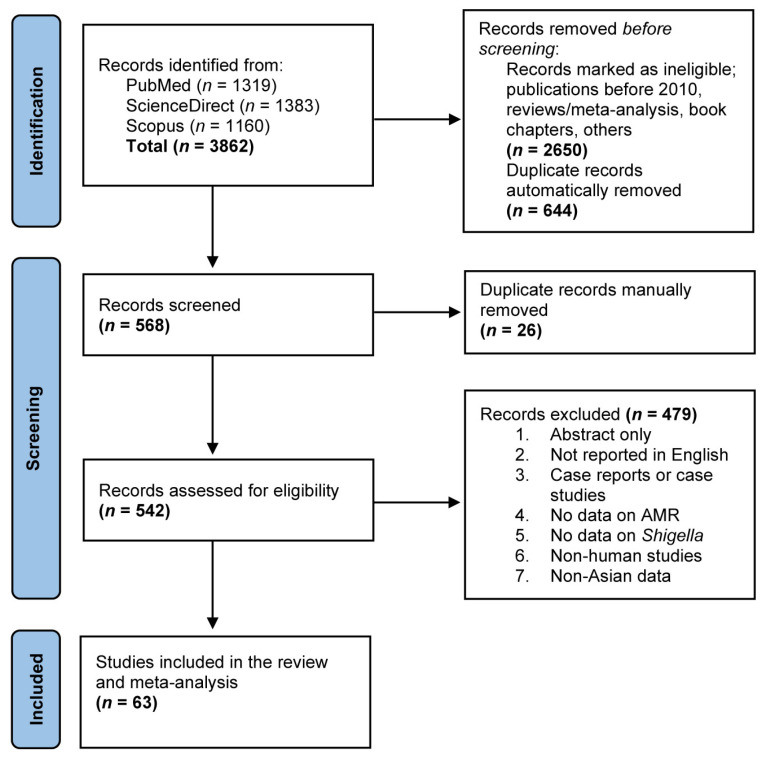
A PRISMA flow diagram of the study selection process and literature search results. Three different web databases (PubMed, ScienceDirect and Scopus) were used to search eligible studies reporting antimicrobial-resistant *Shigella* spp. using predefined search strategies. A total of 3862 records were retrieved and duplicates were removed using EndNote 20 software, following which they were screened against predefined inclusion criteria before inclusion in the qualitative study and meta-analysis.

Figure 1 shows a Preferred Reporting Items for Systematic Reviews and Meta-analyses (PRISMA) flow diagram of our study selection process, as well as results from the literature search. Our online search of three different web databases—namely, PubMed, ScienceDirect and Scopus—returned a total of 3862 records. Following the initial pre-screening eligibility check and duplicate removal, 568 records were screened for their titles and abstracts, of which, after rounds of manual assessment to exclude 26 records, a total of 542 records were retained for full-text evaluation, where they were assessed based on the predetermined exclusion criteria. A total of 479 records were removed and, subsequently, a total of 63 articles were found to be eligible for this study and, thus, were included in the qualitative study and meta-analysis.

**Figure 2:**
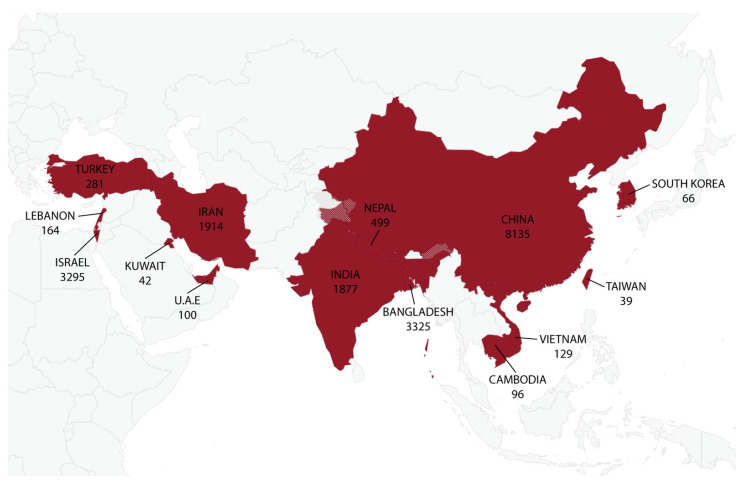
Distribution of *Shigella* spp. across 14 Asian regions from 63 studies. A total of 19,962 cases from the continent were identified involving *Shigella* spp., and China represented the country with the most cases of *Shigella* infections (*n* = 8135), as reported in 15 different studies, whereas the lowest number of cases involving *Shigella* spp. was recorded for Kuwait (*n* = 42) in a single study.

The summarized characteristics of the 63 included articles are shown in Table 1. Studies were conducted in 14 Asian countries, and China represented the country with the most cases of *Shigella* infections *(n* = 8135), as reported in 15 different studies (Figure 2). The country with the second highest number of cases involving *Shigella* spp. was Bangladesh, which recorded 3325 cases in only three studies. Although Iran was represented by 15 studies, the country recorded only 1914 cases involving *Shigella* spp., whereas Israel recorded 3295 cases of *Shigella* infections in just a single study. Overall, a total of 19,962 cases from the Asian region involving *Shigella* spp. and employing antimicrobial susceptibility testing using 49 different antibiotics were identified (Table 1). All 63 included studies were cross-sectional, and they were carried out between 2000 and 2020 and published between 2010 and 2022. *Shigella* isolates were collected mostly from pediatric groups and characterized using polymerase chain reactions (PCRs) or slide agglutination tests (SATs), whereas double-disk synergy tests (DDSTs) were utilized to detect the presence of ESBL genes. Biochemical tests, such as the Kirby–Bauer method (disk diffusion method), broth microdilution, agar dilution and concentration gradient method (E-test), were used in the antibiotic susceptibility tests against the *Shigella* spp. Out of the 63 selected studies, 39 provided data on MDR, whereas only 16 studies provided data on ESBL-producing *Shigella* spp.

### 2.2. Prevalence of *Shigella* species in Asia

**Figure 3:**
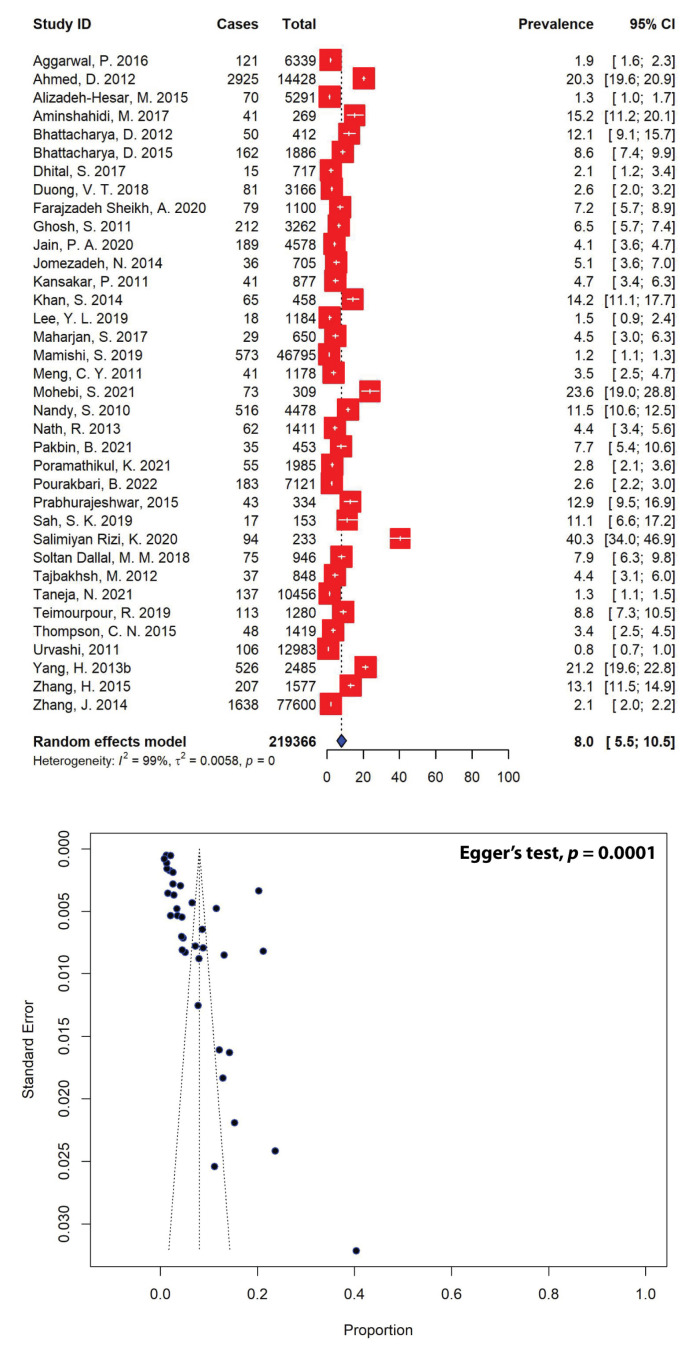
Forest and funnel plots representing the pooled Asian prevalence of *Shigella* spp. from 36 studies. The estimates of pooled prevalence were calculated using the random-effects model (**top panel**). The distribution of effect estimates is represented by a funnel plot (**bottom panel**). Figures were generated using RStudio software.

The pooled prevalence of *Shigella* spp. in Asian patients from 36 studies was estimated to be 8.0% (95% CI: 5.5–10.5) (Figure 3), and the lowest prevalence of *Shigella* spp. in Asia was 0.8%, whereas the highest prevalence of *Shigella* spp. was 40.3%. Of the 63 selected studies, 27 were excluded due to the absence of data on the sample population (Table 1). The asymmetrical distribution of effect estimates, which is shown by a funnel plot of the study distribution (Figure 3), prompted us to examine further the data according to subgroups. When stratified according to the regions where the studies were conducted, the highest estimated *Shigella* spp. prevalence was 10.2% (95% CI: 4.0–16.3), calculated from 12 different studies in the Middle East, followed by the estimates for East Asia (9.5%, 95% CI: 0.2–18.7) and South Asia (7.4%, 95% CI: 4.7–10.2). In contrast, the lowest *Shigella* spp. prevalence of 2.9% (95% CI: 2.5–3.3) was recorded in Southeast Asia. When stratified according to countries, Bangladesh (20.3%, 95% CI: 19.6–20.9), China (12.1%, 95% CI: 1.3–23.0) and Iran (10.2%, 95% CI: 4.0–16.3) represented the three highest *Shigella* spp. prevalence estimates, while Taiwan recorded the lowest *Shigella* spp. prevalence in Asia (1.5%, 95% CI: 0.9–2.4) (Table 2). 

**Figure 4:**
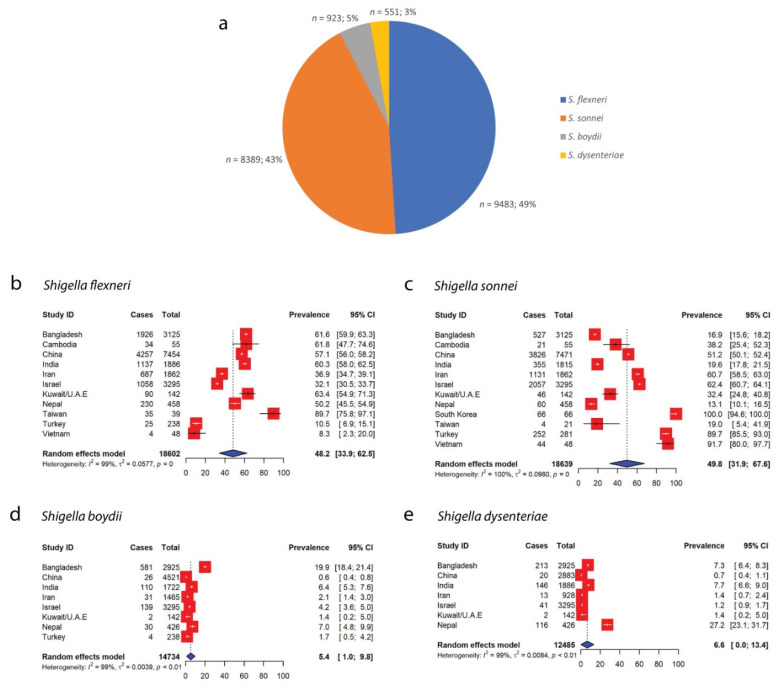
Distribution of different species of *Shigella* across Asian countries. (**a**) Proportions of different species of *Shigella*. *S. flexneri* constituted the highest proportion of *Shigella* isolates in Asia (49%, *n* = 9483), followed by *S. sonnei* (43%), *S. boydii* (5%) and *S. dysenteriae* (3%). (**b**) Forest plot representing the pooled Asian prevalence of *S. flexneri*. (**c**) Forest plot representing the pooled Asian prevalence of *S. sonnei*. (**d**) Forest plot representing the pooled Asian prevalence of *S. boydii*. (**e**) Forest plot representing the pooled Asian prevalence of *S. dysenteriae*.

When grouped according to the species of *Shigella*, the highest numbers of isolates recorded were for *S. flexneri*, with this species accounting for 49% (*n* = 9483) of the 19,346 total cases of *Shigella* infections that were reported in Asia, followed by *S. sonnei* (43%, *n* = 8389), *S. boydii* (5%, *n* = 923) and *S. dysenteriae* (3%, *n* = 551) (Figure 4a). Overall, 19,346 out of 19,962 total *Shigella* isolates were characterized according to their species, whereas five studies [22,23,27,39,45] did not report the species of *Shigella* isolates (Table 1). Although *S. flexneri* accounted for the largest proportion of *Shigella* isolates reported in the included studies, the pooled prevalence of *S. sonnei* was the highest in Asia at 49.8% (95% CI: 31.9–67.6), followed by those of *S. flexneri* (48.2%, 95% CI: 33.9–62.5), *S. dysenteriae* (6.6%, 95% CI: 0.0–13.4) and *S. boydii* (5.4%, 95% CI: 1.0–9.8) (Figure 4). China reported the highest number of cases with *S. flexneri* (*n* = 4257) and *S. sonnei* (*n* = 3826), while the highest cases of *S. boydii* (*n* = 581) and *S. dysenteriae* (*n* = 213) infections were recorded in Bangladesh between 2005 and 2008 [16].

### 2.3. Antimicrobial Resistance Patterns of *Shigella* spp.

The antimicrobial susceptibility levels of the *Shigella* isolates from the 63 included studies were tested against various antibiotics (Table 1). The pooled prevalence estimates of the resistant *Shigella* isolates tested against 49 different antibiotics are presented in Table 3. The antibiotics were classified into 13 groups, with the penicillin group of antibiotics being the most commonly used; in particular, ampicillin was identified as the most commonly used antibiotic (57 studies), while ceftiofur, oxacillin and fosfomycin were the least frequently tested against *Shigella* spp. (one study each) [54,55,64]. Our meta-analysis revealed that resistant *Shigella* strains exist for the majority of the antibiotics tested, although they are varied (Table 3). The antimicrobial drug resistance patterns for *Shigella* spp. in Asia revealed that 98.4% (95% CI: 96.9–100.0) of the isolates were resistant to streptomycin, followed by trimethoprim (95.5%, 95% CI:92.3–98.8) and ticarcillin (90.5%, 95% CI: 84.7–96.3), whereas antimicrobials belonging to the carbapenem class of antibiotics, such as ertapenem (0.0%, 95% CI: 0.0–1.1), meropenem (0.0%, 95% CI: 0.0–0.7) and imipenem (0.1%, 95% CI: 0.0–0.2), had the lowest *Shigella* resistance rate, in addition to nitrofurantoin (0.0%, 95% CI: 0.0–0.2).

### 2.4. Prevalence of Multidrug-Resistant *Shigella* spp. in Asia

**Figure 5:**
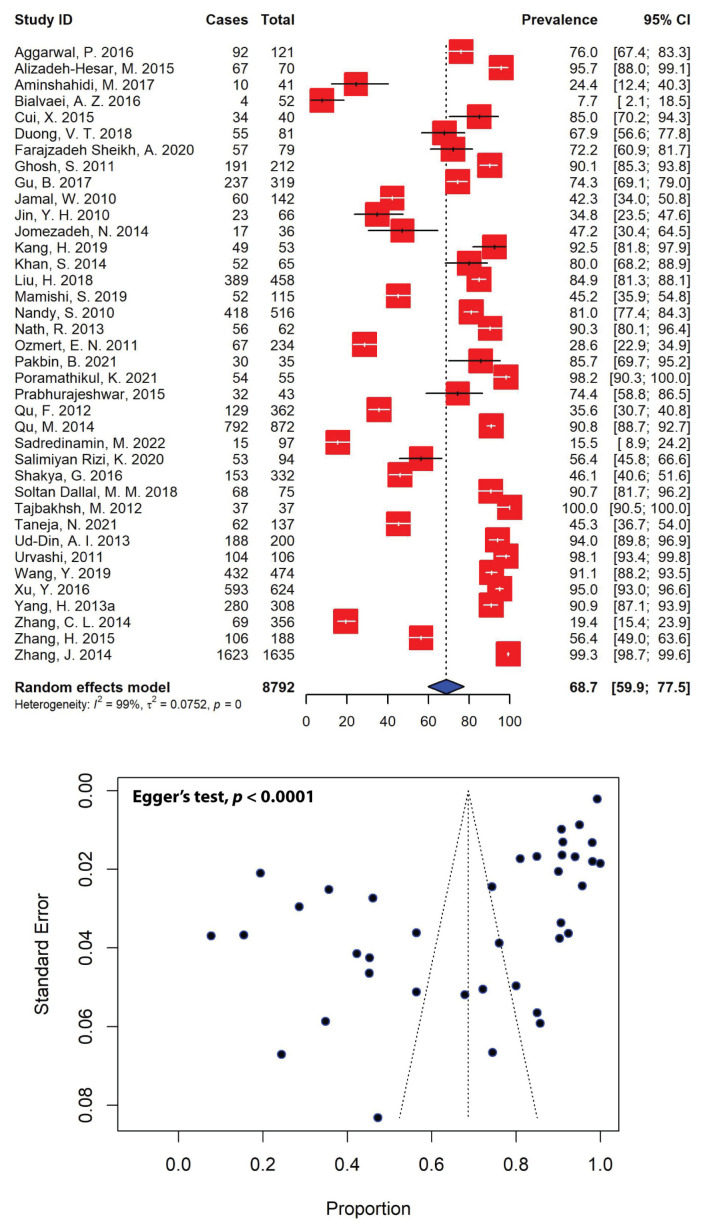
Forest and funnel plots representing the pooled prevalence of multidrug-resistant *Shigella* spp. in Asia. The estimate of prevalence was calculated by pooling 38 selected studies using the random-effects model (**top panel**). The distribution of effect estimates is shown by a funnel plot (**bottom panel**). Figures were generated using RStudio software.

The estimated prevalence of MDR *Shigella* spp. was significantly high across different studies, with the majority (26 out of the 38 included studies) reporting multiple resistance rates higher than 50.0% for total *Shigella* isolates (Figure 5). Our meta-analysis revealed that the pooled prevalence of MDR *Shigella* spp. in Asia was estimated to be 68.7% (95% CI: 59.9–77.5), with evidence of significant heterogeneity (*I*^2^ = 99%, τ^2^ = 0.0752, *p* = 0). The highest and lowest prevalence rates of MDR *Shigella* spp. were estimated to be 100.0% and 7.7%, respectively. The presence of publication bias, represented by an asymmetrical funnel plot (Figure 5), was statistically confirmed with Egger’s test (*p* < 0.0001).

When stratified according to different Asian regions, the highest prevalence of MDR *Shigella* spp. was estimated to be 83.4% (95% CI: 53.8–100.0), which was calculated for Southeast Asia, followed by the estimates for South Asia (77.7%, 95% CI: 66.1–89.3) and East Asia (73.2%, 95% CI: 58.5–87.9). Conversely, the lowest MDR *Shigella* spp. prevalence of 54.3% (95% CI: 38.1–70.4) was reported in the Middle East (Table 4). Overall, a total of 6750 MDR *Shigella isolates* were recorded over a 20-year period (2000–2020). Our meta-analysis revealed that the highest MDR *Shigella* spp. prevalence levels were recorded in Cambodia (98.2%, 95% CI: 90.3–100.0), followed by Bangladesh (94.0%, 95% CI: 89.8–96.9) and India (79.6%, 95% CI: 66.8–92.4), while the lowest prevalence was recorded in Turkey (28.6%, 95% CI: 22.9–34.9). As well as having a *Shigella* spp. prevalence of 76.3% (95% CI: 61.8–90.8), China recorded the highest number of MDR *Shigella* isolates in Asia (*n* = 4733) (Table 4).

### 2.5. Patterns of Extended-Spectrum β-Lactamase-Producing *Shigella* spp.

**Figure 6:**
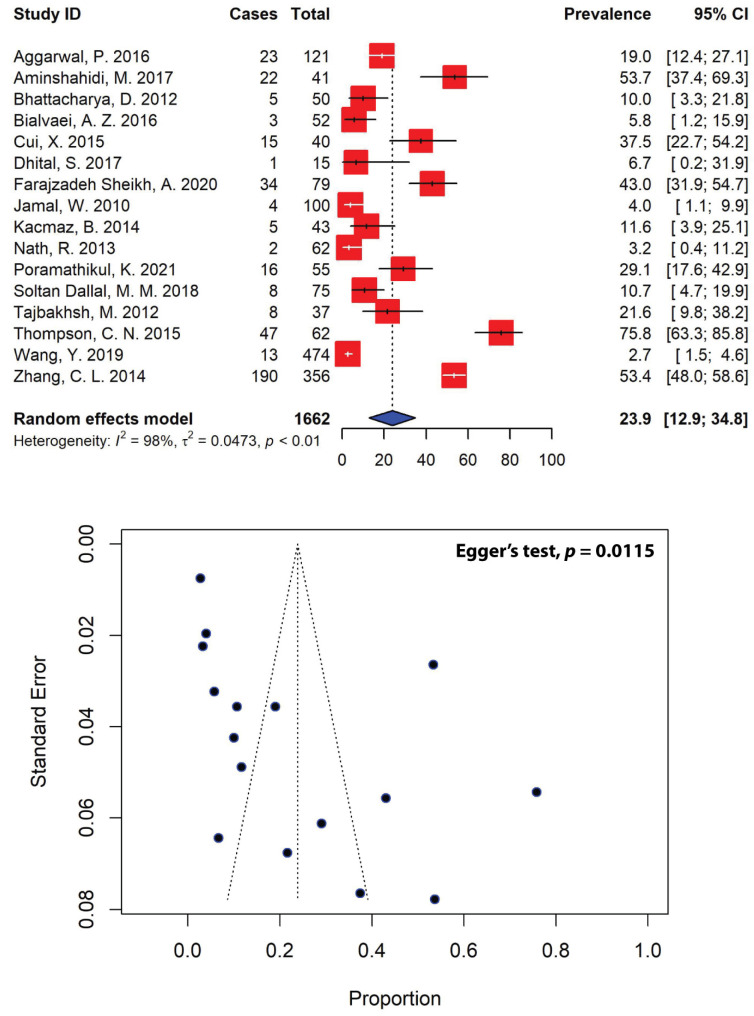
Forest and funnel plots representing the pooled prevalence of extended-spectrum β-lactamase (ESBL)-producing *Shigella* spp. in Asia. The estimate of prevalence was calculated by pooling 16 studies using the random-effects model (**top panel**). The distribution of effect estimates is shown by a funnel plot (**bottom panel**). Figures were generated using RStudio software.

Of the 63 eligible studies analyzed in our meta-analysis, only 16 studies, with a total sample size of 396, reported ESBL-producing *Shigella* spp. in Asian patients (Figure 6). The overall pooled prevalence of ESBL-producing *Shigella* spp. in Asia was estimated to be 23.9% (95% CI: 12.9–34.8), with substantial heterogeneity between studies (*I*^2^ = 98%, τ^2^ = 0.0473, *p* < 0.01). Our meta-analysis revealed that the highest prevalence of ESBL-producing *Shigella* spp. was recorded in a study conducted in Vietnam (75.8%, 95% CI: 63.3–85.8) [67], whereas the lowest prevalence of ESBL-producing *Shigella* spp. was recorded in the U.A.E (4.0%, 95% CI: 1.1–9.9) in a single study [33]. Regionally, Southeast Asia represented the highest prevalence of ESBL-producing *Shigella* spp., with a prevalence of 52.5% (95% CI: 6.8–98.3), followed by East Asia (31.0%, 95% CI: 0.9–61.0), the Middle East (20.7%, 95% CI: 6.7–34.7) and South Asia (9.7%, 95% CI: 2.3–17.1) (Table 5).

## 3. Discussion

*Shigella* continues to represent a significant cause of mortality and morbidity worldwide and has been found to be the second-leading cause of diarrheal mortality among all age groups, accounting for more than 200,000 deaths, including over 63,000 children under the age of 5 years [7]. In a recent global disease burden report, diarrhea was ranked third among the top ten causes of mortality in children younger than 9 years old [77]. Although diarrhea mortality has significantly decreased since 1990, morbidity rates from diarrheal diseases remain high, predominantly in low- and middle-income countries [7,78]. Similarly, shigellosis is a major cause of illness among children, travelers, expatriates and military personnel in low- and middle-income countries, where it is associated with persistent diarrhea of more than 14 days in these populations [7]. However, the global emergence of antimicrobial-resistant *Shigella* strains, which limit the choice of effective drugs for shigellosis, has become the main challenge in the treatment of *Shigella* infections. Moreover, changes in the prevalent *Shigella* serogroups/types and resistance patterns are creating a major burden for the choice of an appropriate therapy for shigellosis treatment. The distribution of *Shigella* serogroups/types has varied over different periods of time and in different geographical regions. For instance, *S. flexneri* serotypes such as 2a, 3a and 1a, are most prevalent in Asian countries such as China, India and Pakistan [79]. Even though *S. sonnei* is traditionally most commonly found in developed countries, the species is currently dominant and undergoing an unprecedented expansion across developing countries in Asia, Latin America and the Middle East [3]. A previous study reported that *Shigella* infections increase during the monsoon and summer seasons, when the humidity and temperature are high [65]. *S. flexneri*, together with *S. sonnei*, are responsible for more than 90% of the global shigellosis cases [2]. Although shigellosis is mostly a self-limiting disease, antibiotics are recommended to reduce deaths and disease progressions. However, in recent years, *Shigella* has been reported to be becoming resistant to many antimicrobial drugs, such as ampicillin, tetracyclines, chloramphenicol, ciprofloxacin, nalidixic acid and trimethoprim/sulfamethoxazole [79,80]. This phenomenon can be seen in many regions across the globe [2,28,29,30,81,82,83]. Therefore, determining the *Shigella* burden in a population is vital for the design of targeted therapeutic strategies to reduce the incidence of mortality and morbidity from shigellosis. This requires comprehensive data on the prevalence and patterns of drug-resistant *Shigella* spp. but, to the best of our knowledge, such data are unavailable to date in Asia.

Here, we present the prevalence of multidrug-resistant and extended-spectrum beta-lactamase-producing *Shigella* spp. in Asia. Our findings in this SRMA were calculated by combining all eligible data on the prevalence of antimicrobial-resistant *Shigella* spp. from community- and hospital-based studies, as reported in the 63 selected studies from Asia. Nevertheless, as expected from various studies with different backgrounds and settings, our findings were mostly heterogeneous. Significant heterogeneity was also observed in our previous study on the prevalence of multidrug-resistant diarrheagenic *Escherichia coli* (DEC) [84]. The heterogeneity in our findings was most likely due to the fact that different sample sizes, methodologies and research settings—such as study regions, time periods and population ages—were employed in different studies. This is expected, as our SRMA utilized a random-effects model, which presumes heterogeneity, as opposed to being a meta-analysis using a fixed-effects model [85]. Nonetheless, to the best of our knowledge, our SRMA is the first to evaluate the prevalence of antimicrobial-resistant *Shigella* spp. in Asia and, thus, it will hopefully be useful for designing targeted strategies in the treatment of shigellosis.

The pooled prevalence estimates revealed that 8.0% (95% CI: 5.5–10.5) of all Asian diarrheal cases in the last couple of decades were caused by *Shigella* (Figure 3), and the highest and lowest prevalence estimates for *Shigella* were 40.3% [60] and 0.8% [68], respectively. Iran had the highest number of studies reporting the prevalence of *Shigella* among those analyzed in our SRMA, accounting for 12 different studies (Table 2). The pooled prevalence of *Shigella* in Iran was estimated at 10.2% (95% CI: 4.0–16.3), slightly higher than the overall pooled estimate for *Shigella* in Asia. Nevertheless, the pooled prevalence of *Shigella* calculated in our SRMA was higher than the results of a similar comprehensive prevalence estimate from Iran, for which an estimate of 6.2% was calculated [86]. Differences in the pooled prevalence estimates between our study and the recently published study might be attributed to differences in the numbers of included studies, sample sizes, study settings and methods, publication years and many other factors. It is likely that the prevalence of *Shigella* calculated in this SRMA was higher due to the smaller number of included studies (*n* = 12), whereas there were 34 included studies, published in English and Persian from 2000 to 2020, in the previous comprehensive review [86]. The pooled prevalence estimate for *Shigella* in India was calculated at 6.2% (95% CI: 3.4–9.0)—lower than the overall prevalence of Shigella in Asia—from 10 included studies (Table 2), whereas a prevalence of 12.1% (95% CI: 1.3–23.0) was estimated for China from three included studies. It is important to note that, although China was represented by only three studies, the country had the largest sample population of 81,662, which constituted 37.2% of the total diarrheal Asian population analyzed in this meta-analysis. Taiwan had the smallest sample population of 1184, with the prevalence estimate for *Shigella* in the country being calculated at 1.5% (95% CI: 0.9–2.4) [41]—the lowest in Asia. In contrast, Bangladesh had the highest prevalence of *Shigella* in Asia, which as estimated at 20.3% (95% CI: 19.6–20.9) from a single study conducted in Dhaka [16].

Historically, *S. flexneri* is known to be commonly found in developing countries, while *S. sonnei* is traditionally most commonly isolated in developed countries. However, in recent years, *S. sonnei* has become increasingly dominant and can be found in many developing countries across the globe. Our pooled prevalence estimates revealed that *S. sonnei* had the highest prevalence of 49.8% (95% CI: 31.9–67.6) in Asia, followed by *S. flexneri* (48.2%, 95% CI: 33.9–62.5), *S. dysenteriae* (6.6%, 95% CI: 0.0–13.4) and *S. boydii* (5.4%, 95% CI: 1.0–9.8) (Figure 4). While South Korea had the highest prevalence of *S. sonnei* in Asia, the species has now become dominant in developing countries, such as Iran, Israel, Turkey and Vietnam, where it was estimated at 60.7%, 62.4%, 89.7% and 91.7% of the total *Shigella* spp. isolated from the respective countries (Figure 4c). *S. flexneri*, conversely, was found to still be dominant in many developing countries, such as Bangladesh, Cambodia, China, India, Kuwait/U.A.E and Nepal (Figure 4b). Although Taiwan is regarded as a highly developed country, *S. flexneri* was found to have the highest prevalence (89.7%, 95% CI: 75.8–97.1) in the country, followed by *S. sonnei* (19.0%, 95% CI: 5.4–41.9). The prevalence estimates of *S. boydii* and *S. dysenteriae*, on the other hand, were the highest in Bangladesh (19.9%, 95% CI: 18.4–21.4) and Nepal (27.2%, 95% CI: 23.1–31.7), respectively. Our estimated prevalence for *S. sonnei* in Iran was slightly higher than previously reported, where it was estimated to be 54.1%, while our pooled prevalence for *S. flexneri* was slightly lower (36.9% vs. 40.1%) [86]. In an SRMA published in 2012, it was reported that the prevalence of *S. sonnei* in China was estimated to be 21.3% [87]. However, here we report a significant increase in the prevalence of *S. sonnei* in that country, estimated to be 51.2%. While it was previously reported that the prevalence of *S. flexneri* in China was estimated to be 76.2% [87], our analysis showed a substantial decrease in the prevalence of the species in that country (Figure 4b). Increases in the prevalence of *S. sonnei* in both countries could be attributable to the unprecedented global spread of the species across developing countries in Asia, Latin America and the Middle East [3].

Concerning the antimicrobial drugs used to treat shigellosis, the WHO published guidelines in 2005 for shigellosis treatment, with a recommendation of ciprofloxacin as the first-line antimicrobial therapy for all patients with bloody diarrhea, irrespective of age. In cases of multi-resistant strains of *Shigella* with resistance against ciprofloxacin, pivmecillinam and ceftriaxone were recommended as the second-line treatment in all age groups, while azithromycin was also recommended as an alternative for treatment of adults [8,88]. Antibiotics such as nalidixic acid, ampicillin, amoxicillin, chloramphenicol, sulfamethoxazole/trimethoprim and tetracyclines were highlighted as ineffective against *Shigella* due to increasing antimicrobial resistance [8]. These antibiotics were commonly used in the past to treat shigellosis. In this meta-analysis, resistance against all classes of antibiotics was common among *Shigella* isolates, except for carbapenems, against which resistance was almost non-existent (Table 3). The pooled prevalence of *Shigella* resistant against streptomycin was the highest, estimated at 98.4% (95% CI: 96.9–100.0), followed by trimethoprim (95.5%, 95% CI: 92.3–98.8) and ticarcillin (90.5%, 95% CI: 84.7–96.3). High resistance rates were observed against the penicillin class of antibiotics, with more than 70% of *Shigella* isolates being resistant to amoxicillin (73.2%, 95% CI: 52.4–94.1), ampicillin (72.6%, 95% CI: 66.4–78.7) and oxacillin (71.9%, 95% CI: 53.3–86.3). While the prevalence of *Shigella* isolates resistant to penicillins was relatively high, combinations with other antimicrobial agents somehow decreased the resistance to amoxicillin, ampicillin, piperacillin and ticarcillin. For instance, the combination of amoxicillin with clavulanic acid rendered *Shigella* more susceptible, with the pooled prevalence of resistant *Shigella* decreasing from 73.2% to 23.4% (95% CI: 12.4–34.4). Similarly, the combination of ampicillin with sulbactam decreased the resistance of *Shigella* from 72.6% to 27.5% (95% CI: 15.1–39.9). It is possible that utilizing more than one β-lactam antibiotic in the treatment of shigellosis decreases the resistance of the pathogen significantly.

Relatively high proportions of *Shigella* isolates were resistant to ciprofloxacin (29.8%, 95% CI: 22.4–37.1), mecillinam (37.8%, 95% CI: 8.8–66.7), ceftriaxone (23.8%, 95% CI: 16.1–31.6) and azithromycin (29.2%, 95% CI: 20.8–37.6), in spite of their importance as first- and second-line treatments for shigellosis. High rates of resistance to these antibiotics are unfortunate developments in the majority of Asian countries and could reflect the excessive and unjustified use of antibiotics in general care to treat shigellosis. In India, 67.9% of the total *Shigella* isolates were reported to be resistant to ciprofloxacin, and 60.2% of *S. flexneri* and 57.6% of *S. dysenteriae* were found to be resistant to the antibiotic between 2004 and 2008 [68]. In a more recent study published in 2021, resistance to ciprofloxacin was recorded in 61.5% of the total *Shigella* isolates in the country, and resistance rates in *S. flexneri* and *S. sonnei* isolates were reported at 63.9% and 58.3%, respectively [65]. A study in China found that the rate of resistance against ciprofloxacin was comparatively low, recorded at 11.6% of the total *Shigella* isolates in the past decade [75], while another study reported a higher rate of resistance of 27.9% against the antibiotic within the same period [71]. A total of 25.4% of *S. flexneri* and 35.0% of *S. sonnei* were found to be resistant to ciprofloxacin in China between 2005 and 2011 [71]. Additionally, findings of high resistance against first-generation cephalosporins, such as cephalothin (33.6%, 95% CI: 10.7–56.5) and cefalexin (56.4%, 95% CI: 0.0–100.0); kanamycin (51.9%, 95% CI: 0.8–100.0); and erythromycin (57.4%, 95% CI: 51.6–63.2) are alarming, as we are now left with a limited number of effective antibiotics for the treatment of shigellosis. 

Resistance in *Shigella* spp. against a wide array of antimicrobial drugs was also reported by a comprehensive study in Ethiopia, where high resistance rates against ampicillin (83.1%), amoxicillin (84.1%) and erythromycin (86.5%) were recorded, while comparably low resistance was reported for ciprofloxacin (8.9%), ceftriaxone (9.3%), norfloxacin (8.2%) and gentamycin (17.3%) [82]. In another report, rates of resistance to nalidixic acid and ciprofloxacin in Asia–Africa were 33.6% and 5.0%, respectively, but progressively increased each year, reaching 64.5% and 29.1%, respectively, in 2007–2009. In contrast, rates of resistance to nalidixic acid and ciprofloxacin in Europe–America remained low, estimated at 2.1% and 0.6%, respectively [2]. As resistance to ciprofloxacin and ceftriaxone continues to increase each year, the use of both antibiotics empirically as the first-line antimicrobial therapy in the treatment of shigellosis should be reconsidered. Due to the distribution of different *Shigella* spp. and variability in their antibiotic resistance profiles from one geographical location to another, which may also change with time, continuous assessment of resistance patterns is necessary for appropriate treatment of shigellosis. Nevertheless, our results showed that resistance to carbapenems, such as ertapenem (0.0%, 95% CI: 0.0–1.1), imipenem (0.1%, 95% CI: 0.0–0.2) and meropenem (0.0%, 95% CI: 0.0–0.7), was the lowest in Asia among the 49 tested antibiotics. Although the resistance rates against the carbapenem antibiotics were almost non-existent, careful and justified use of such antibiotics is essential to limit the emergence of new drug-resistant strains.

**Figure 7:**
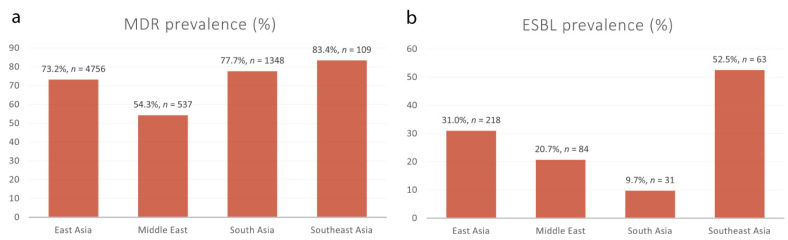
The prevalence rates of MDR and ESBL-producing *Shigella* isolates grouped according to different Asian regions. (**a**) The prevalence rates of MDR *Shigella* spp. in East Asia (73.2%), the Middle East (54.3%), South Asia (77.7%) and Southeast Asia (83.4%). (**b**) The prevalence rates of ESBL-producing *Shigella* spp. in East Asia (31.0%), the Middle East (20.7%), South Asia (9.7%) and Southeast Asia (52.5%).

Furthermore, the high resistance burden of many antimicrobial drugs has resulted in relatively high rates of MDR in *Shigella* isolates. Evidence suggests that MDR is an alarming challenge in the treatment of shigellosis, with about half of *Shigella* strains around the globe now being resistant to multiple antibiotics [13]. For instance, *S. sonnei* isolates carrying the plasmid-mediated polymyxin resistance gene *mcr-1* have been shown to be resistant to azithromycin and third-generation cephalosporins, including ceftriaxone and cefoperazone [89]. Resistance to third-generation cephalosporins has also been linked to the production of ESBLs, conferring resistance to β-lactamases [90]. Our pooled estimate of MDR *Shigella* in Asia was 68.7% (95% CI: 59.9–77.5) (Figure 5)—substantially higher than that in a report from Brazil (43.8%) [91], but much lower than reports from Egypt (88.0%) [92] and Ethiopia (83.2%) [82]. The highest prevalence of MDR *Shigella* spp. was recorded in Southeast Asia, which was estimated to be 83.4%, followed by South Asia (77.7%), East Asia (73.2%) and the Middle East (54.3%) (Figure 7). This is alarming, as *Shigella* resistance to multiple antibiotics in the Asian population has been steadily increasing in the past few decades, especially resistance against quinolones, penicillins and third-generation cephalosporins. Multiple drug-resistant pathogens are currently considered to be significant global public health threats. For example, the pooled prevalence of MDR *Pseudomonas aeruginosa* in Spain was reported at 5.0%, while MDR *Acinetobacter baumanii*, *Klebsiella pneumoniae* and *E. coli* were reported at 10.0%, 32.5% and 40.0%, respectively [93]. Among diarrheagenic pathogens in Ethiopia, the highest prevalence of 80.8% was recorded for MDR *Campylobacter*, followed by MDR *E. coli* and *Salmonella*, recorded at 78.2% and 59.5%, respectively [94]. In Asia, the pooled prevalence of MDR-DEC was estimated to be 66.3% (95% CI: 58.9–73.7) [84]. The acquisition of ESBL genes is one of many mechanisms that could lead to an increase in MDR *Shigella*. In our analysis, the prevalence of ESBL-producing *Shigella* spp. in Asia was 23.9% (95% CI: 12.9–34.8) (Figure 6). The highest prevalence of ESBL-producing *Shigella* spp. was recorded in Southeast Asia (52.5%), followed by East Asia (31.0%), the Middle East (20.7%) and South Asia (9.7%) (Figure 7). In Australia, there was a significant increase in cases of ESBL-producing *S. sonnei* in the recent years, with 65% of *S. sonnei* isolates carrying the *bla*_CTX-M-27_ gene, which confers resistance to extended-spectrum cephalosporins. These isolates were multidrug-resistant, including resistance to azithromycin and co-trimoxazole, and had reduced susceptibility to ciprofloxacin [95]. In a study of the Dutch population, the prevalence of ESBL-producing Enterobacteriaceae was estimated to be 10.1%, and the most common isolate was CTX-M-15, accounting for 47% of the ESBL-producing organisms in the country. This is worrying; even though the rate of antibiotic consumption in humans is low in the Netherlands, due to the spread of CTX-M ESBLs, particularly CTX-M-15, resistance is emerging [96].

Our SRMA is the first to provide a comprehensive analysis of antimicrobial-resistant *Shigella* spp. in the Asian population. However, it has several notable limitations. First, even though a significant number of studies were included (*n* = 63) in this SRMA, they did not incorporate all of the countries in Asia; thus, the estimated prevalence might not wholly represent the true scale of antimicrobial-resistant *Shigella* spp. in Asia. Nevertheless, data were collected from 14 Asian countries—a large number of shigellosis cases (*n* = 19,962) were analyzed. Second, significant heterogeneity was observed in our SRMA, although this is common in meta-analyses of prevalence estimation [82,84,87,94,97]. It was expected, as we utilized a random-effects model in our meta-analysis, which presumes heterogeneity [85]. Third, the potential effects of gender and age distribution on the prevalence of *Shigella* spp. could not be accounted for in this study due to the nature of the data reporting in most of the included studies; while some studies reported antimicrobial resistance from pediatrics, others reported data from various age groups, and many did not disclose the patient age groups (Table 1). The last and most important limitation is that the patterns of multidrug resistance of different *Shigella* spp. could not be obtained owing to the different types of data presented in various studies. Many of the included studies did not report the prevalence of antimicrobial resistance of different *Shigella* spp. but instead reported the prevalence of drug-resistant *Shigella* collectively. Thus, we were also unable to account for the prevalence of antimicrobial-resistant *Shigella* on a yearly basis. Nevertheless, we believe that such crucial information is needed and would be helpful to clinicians, researchers and governments.

## 4. Methodology

### 4.1. Literature Search Strategy and Selection

This SRMA was conducted based on the guidelines of the Preferred Reporting Items for Systematic Reviews and Meta-analyses (PRISMA) [98]. The protocol of this study was registered in the International Prospective Register of Systematic Reviews (PROSPERO) database (registration number: CRD42022352131). From July 2022 to August 2022, a comprehensive literature search was performed to find eligible studies on the prevalence of antimicrobial-resistant *Shigella* spp. in Asia that were available in the PubMed, ScienceDirect and Scopus databases (Figure 1). All eligible articles were retrieved from the three databases using relevant search terms and keywords, which included “*Shigella sonnei* AND drug resistance AND Asia”, “*Shigella flexneri* AND drug resistance AND Asia”, “*Shigella boydii* AND drug resistance AND Asia”, “*Shigella dysenteriae* AND drug resistance AND Asia” and “*Shigella* AND drug resistance AND Asia”. In addition, the reference lists of the selected articles were checked for more related eligible articles.

### 4.2. Inclusion and Exclusion Criteria 

Only studies that reported sufficient data for determining the prevalence of antimicrobial-resistant *Shigella* spp. in patients from countries in the Asian region, regardless of gender and age, were considered eligible for inclusion in this SRMA. Additionally, articles that reported the prevalence of ESBL-producing *Shigella* spp. were also included in this study. Among such articles, only full-length original research articles written in the English language were considered for the analysis. Furthermore, only articles that were published from 2010 to July 2022 were selected in order to obtain up-to-date information on the topic. Studies that did not report any data on *Shigella* spp. and their antimicrobial susceptibility, case reports and case studies, review articles and short communications, as well as studies with abstracts only, were excluded from the analysis. Moreover, unpublished articles or articles with incomplete information, as well as all articles published before 2010, were not included in the study. Only data from human-related studies were included in the analysis.

### 4.3. Data Extraction and Quality Control

All eligible studies were collected and managed using EndNote 20, in which duplicate articles were removed, and the remaining articles were assessed systematically based on the titles and abstracts. Two authors, M.Z.S. and N.M.N.N.Z., independently evaluated the eligibility of all articles by examining the full texts using predetermined inclusion criteria. Only 63 eligible articles were selected and coded, and data from each article were extracted to a table in Microsoft Excel that contained necessary information, such as the author names, title, year of publication, study period, study region, study design and methods used, sample population, sample size, sample type, age group, gender, the isolated *Shigella* spp., resistance patterns of the *Shigella* isolates and the prevalence of MDR and ESBL-producing *Shigella* spp. In this study, MDR was defined as resistance to three or more antimicrobial drugs.

### 4.4. Data Analysis

All 63 selected studies were included in the meta-analysis, and the analysis was carried out using metaprop codes in the meta (version 5.2-0) and metafor (version 3.4-0) packages of R (version 4.2.1), as implemented in RStudio (Posit, PBC, Boston, USA) (version 2022.02.2+485) [99]. The pooled prevalence of *Shigella* spp. resistant to each antibiotic, the pooled prevalence of MDR and ESBL-producing *Shigella* isolates and 95% confidence intervals (CIs) were calculated using the REML method for the random-effects model. In addition, statistical heterogeneity between the studies was measured using Cochran’s Q test for the heterogeneity significance and inconsistency index (*I*^2^) [100], for which *I*^2^ of >75% and a significance level of <0.05 (*p*-value) were interpreted as evidence of significant heterogeneity. The presence of publication bias was determined by evaluating a funnel plot, with significance tested using Egger’s test only for the included studies with publication bias greater than 10.

## 5. Conclusions

Our SRMA presents significant evidence of antimicrobial-resistant *Shigella* spp. distribution in the Asian population. The pooled prevalence estimates showed a substantially high proportion of multidrug-resistant *Shigella* in Asia, posing a major burden to public health. Our meta-analysis revealed that the pooled prevalence of MDR *Shigella* in Asia was estimated to be 68.7%, substantially higher than in many other countries, while the pooled prevalence of ESBL-producing *Shigella* strains was 23.9%. Even though the prevalence was estimated to vary across different Asian countries, the evidence suggests that multidrug resistance is a pressing threat to public health worthy of careful and justified interventions. It is therefore vital to continuously monitor antimicrobial-resistant *Shigella* spp. by conducting vigorous drug susceptibility tests so that reliable and effective antibiotic resistance mitigation strategies can be implemented, which may lead to better outcomes for the treatment and control of shigellosis in Asia, as well as in different parts of the world.

## Figures and Tables

**Table 1:** Major characteristics of the 63 studies included in this systematic review and meta-analysis (SRMA).

No	Study ID (Author, Year)	Ref.	Study Period	Country	Sample Population	Age	Gender	Sample Size	Bacterial Species	Study Methods	Tested Antibiotics ^1^
1	Aggarwal, P., 2016	[15]	2009 to 2012	India	6339	All age groups	ND	121	SF, SB, SS, SD	SAT, KB, PCR, DDST	AMP, SAM, GEN, AMK, DOX, CHL, SXT, NAL, CIP, OFX, CTR, CAZ, CTX, TZP, IMI, MEM, ETP
2	Ahmed, D., 2012	[16]	2005 to 2008	Bangladesh	14,428	All age groups	ND	2925	SF, SB, SS, SD	KB	AMP, CIP, NAL, SXT
3	Alizadeh-Hesar, M., 2015	[17]	11/2012 to 10/2013	Iran	5291	0–5 years	ND	70	SS, SF, SB	SAT, PCR, KB	STR, SXT, TET, MIN, GEN, NAL, CIP
4	Aminshahidi, M., 2017	[18]	8/2014 to 2/2015	Iran	269	0–18 years	141 M, 128 F	41	SF, SS	KB, PCR	MEM, CAZ, CTX, CTR, CIP, AMK, AMP, SXT, GEN
5	Behruznia, P., 2022	[19]	3/2017 to 9/2019	Iran	ND	0–14 years	72 M, 48 F	120	SS, SF, SB	SAT, PCR, KB	AMP, NAL, CTX, CFM, MIN, CIP, LEV
6	Bhattacharya, D., 2015	[20]	2000 to 2011	India	1886	0–14 years	1245 M, 641 F	162	SF, SB, SS, SD	KB, DDST	AMP, NAL, TET, NOR, SXT, CIP, OFX, CAR, AZI, AMK, CEP, CHL, CFX, GEN, CFM, CTR, CTX, CAZ, AMC, IMI
7	Bhattacharya, D., 2012	[21]	2006 to 2009	India	412	6 months to 14 years	ND	50	SF, SS, SD	KB, E-test, DDST	AMP, NAL, TET, CIP, OFX, SXT, NOR, CAR, AZI, AMK, CHL, GEN, LEX, CFX, CFM, CTR, CTX, CAZ, AMC, IMI
8	Bialvaei, A. Z., 2016	[22]	1/2012 to 12/2013	Iran	ND	ND	ND	52	*Shigella*	KB, PCR, E-test, DDST	AMP, AMX, CEP, GEN, CAZ, TET, CIP, IMI
9	Chamoun, K., 2016	[23]	1/2011 to 12/2013	Lebanon	ND	ND	ND	164	*Shigella*	KB, DDST	AMP, AMC, CTX, CTR, CPM, SXT, CIP, NOR
10	Cui, X., 2015	[24]	2005 to 2013	China	ND	0–4 years	ND	40	SF	Broth microdilution, PCR	CAZ, CTR, CPM, CFP, CFZ, FOX, IMI, PIP, AMP, TIC, TET, TOB, GEN, AMK, AZT, CHL, TCC, LEV, NOR, SXT
11	Das, A., 2016	[25]	2012 to 2015	India	ND	ND	ND	184	SF, SB, SS, SD	KB, E-test, SAT, PCR	CIP, LEV, OFX
12	Dhital, S., 2017	[26]	1/2014 to 12/2014	Nepal	717	0–5 years	ND	15	SF, SS	KB, SAT, DDST, Agar dilution	AMX, NAL, SXT, CIP, TET, AZI, CFM, CAZ, CTR, SAM
13	Duong, V. T., 2018	[27]	5/2014 to 4/2016	Vietnam	3166	1 month to 15 years	1945 M, 1221 F	81	*Shigella*	KB	NAL, CIP, CTR, CAZ, AMC, AMP, SXT, AZI, CHL, IMI
14	Farajzadeh Sheikh, A., 2020	[28]	3/2019 to 1/2020	Iran	1100	0–15 years	650 M, 450 F	79	SF, SS	SAT, KB, PCR, DDST	IMI, SXT, AMP, FOX, CTR, CAZ, CTX, CPM, ERY, CIP, AMK, GEN
15	Ghosh, S., 2011	[29]	11/2007 to 10/2010	India	3262	All age groups	ND	212	SF, SB, SS, SD	SAT, PCR, KB	SXT, NAL, TET, CIP, NOR, OFX, CHL, AMP, AZI, CTR
16	Gu, B., 2017	[30]	2006 to 2011	China	ND	ND	ND	319	SS	SAT, KB, PCR	AMP, AMC, CEP, CTX, GEN, NAL, NOR, TET, SXT
17	Iqbal, M. S., 2014	[31]	2006 to 2011	Bangladesh	ND	ND	ND	200	SF	KB, PCR, Agar dilution	SXT, NAL, AMP, ERY, TET, CIP, CTR
18	Jain, P. A., 2020	[32]	1/2006 to 12/2017	India	4578	All age groups	110 M, 79F	189	SF, SB, SS, SD	KB, SAT	AMP, SXT, CIP, CTR
19	Jamal, W., 2010	[33]	4/2003 to 3/2005	Kuwait	ND	ND	ND	42	SF, SB, SS, SD	E-test, SAT	AMK, AMP, AMC, CTX, CTR, CFX, CHL, CIP, GEN, IMI, MEM, TZP, TET, SXT
			1/2003 to 12/2004	United Arab Emirates	ND	ND	ND	100			
20	Jean, S. S., 2018	[34]	1/2017 to 12/2017	Taiwan	ND	ND	ND	21	SF, SS	Broth microdilution, PCR	AMP, CTR, ETP, CIP, LEV, SXT, AZI
21	Jin, Y. H., 2010	[35]	1999 to 2008	South Korea	ND	ND	ND	66	SS	KB, PCR	AMP, SAM, CEP, STR, KAN, CTR, CIP, CHL, GEN, NAL, TET, SXT
22	Jomezadeh, N., 2014	[36]	6/2011 to 5/2013	Iran	705	0–5 years	313 M, 392 F	36	SF, SB, SS, SD	KB, SAT	AMP, TET, SXT, NAL, CTR, CHL, CIP, CFM, GEN
23	Kacmaz, B., 2014	[37]	2004 to 2006	Turkey	ND	5–75 years	23 M, 20 F	43	SS	KB, PCR, DDST	AMP, CHL, CIP, SAM, AMC, CTR, CFM, SXT
24	Kang, H., 2019	[38]	12/2010	China	ND	ND	ND	53	SF, SS	KB, SAT, PCR	CAZ, AZT, AMP, AMX, PIP, CPM, AMC, IMI, MEM, MIN, CHL, TET, GEN, CTX, CIP, LEV, SXT
25	Kansakar, P., 2011	[39]	1/2002 to 12/2004	Nepal	877	All age groups	ND	41	*Shigella*	KB	AMP, CIP, NAL, SXT, CTR, MEC
26	Khan, S., 2014	[40]	9/2011 to 3/2013	Nepal	458	All age groups	36 M, 39 F	65	SF, SB, SS, SD	KB, SAT	AMK, AMP, AMC, CTX, CAZ, CTR, CHL, CIP, SXT, DOX, GEN, IMI, NAL, NOR, OFX
27	Lee, Y. L., 2019	[41]	2018	Taiwan	1184	ND	ND	18	SF	Broth microdilution, PCR	SAM, CTX, CAZ, CPM, TZP, ETP, IMI, MEM, CIP, LEV, SXT
28	Liu, H., 2018	[42]	2008 to 2014	China	ND	All age groups	252 M, 206 F	458	SF, SS	Broth microdilution, PCR	AMK, AMP, AZT, CFZ, CPM, CFP, FOX, CAZ, CTR, CHL, GEN, IMI, LEV, NIT, NOR, PIP, SXT, TET, TIC, TCC, TOB
29	Maharjan, S., 2017	[43]	6/2014 to 12/2014	Nepal	650	All age groups	28 M, 22 F	29	SF, SB, SS, SD	KB, SAT	AMP, SXT, NAL, CTX, CHL, CIP, OFX
30	Mamishi, S., 2019	[44]	3/2011 to 3/2016	Iran	46,795	3–7 years	290 M, 283 F	573	SF, SB, SS, SD	KB, SAT	AMP, CTX, SXT, NAL, GEN, AMK, CIP
31	Meng, C. Y., 2011	[45]	11/2004 to 10/2006	Cambodia	1178	3 months to 5 years	664 M, 514 F	41	*Shigella*	KB	AZI, ERY, NAL, CIP, AMP, GEN, SXT, TET
32	Mohebi, S., 2021	[46]	10/2018 to 3/2019	Iran	309	0–18 years	162 M, 147 F	73	SF, SS, SB	KB, SAT, PCR	AMP, SXT, NAL, CTR, CTX, AZI, CFM, AZT, CHL, CAZ, CIP, SAM, AMK, IMI, GEN
33	Nandy, S., 2010	[47]	1/2001 to 12/2007	India	4478	0–5 years	273 M, 243 F	516	SF, SB, SS, SD	KB, SAT	AMP, TET, CHL, SXT, NAL, CIP, NOR, GEN, AMK, CTX, OFX
34	Nath, R., 2013	[48]	1/2008 to 11/2010	India	1411	All age groups	ND	62	SF	KB, SAT, DDST	AMP, SXT, TET, CHL, NAL, CIP, NOR, OFX, CTR, AZI, CTX, IMI, GEN, AMK, AMC, SAM, FUR, TZP
35	Ozmert, E. N., 2011	[49]	2003 to 2009	Turkey	ND	All age groups	136 M, 102 F	238	SF, SS, SB	KB, SAT	SXT, NAL, AMP, CIP
36	Pakbin, B., 2021	[50]	11/2019 to 7/2020	Iran	453	2–5 years	242 M, 211 F	35	SF, SS, SB	KB, PCR	IMI, AMP, TET, AMK, CHL, NAL, AZI
37	Peleg, I., 2014	[51]	2005 to 2009	Israel	ND	ND	ND	3295	SF, SB, SS, SD	KB	AMP, SXT, NAL, CTR, OFX
38	Poramathikul, K., 2021	[52]	7/2014 to 6/2019	Cambodia	1985	All age groups	ND	55	SF, SS	KB, DDST	AMP, AZI, CTX, CAZ, CTR, CIP, NAL, SXT, TET
39	Pourakbari, B., 2022	[53]	9/2018 to 2/2020	Iran	7121	1–16 years	93 M, 90 F	183	SF, SS	KB, PCR	NAL, AMK, AMP, GEN, CTX, CIP, SXT
40	Prabhurajeshwar,, 2015	[54]	7/2013 to 3/2014	India	334	1 month to 5 years	26 M, 17 F	43	SF, SS, SD	KB	AMP, AMK, GEN, IMI, TET, LEX, CPM, CIP, CTR, SXT, CAZ, NAL, CTX, CHL, OFX, OXA
41	Qu, F., 2012	[55]	1/2002 to 12/2007	China	ND	All age groups	197 M, 165 F	362	SS	KB, SAT	AMP, PIP, CTR, CPM, CIP, NOR, OFX, LEV, CHL, SXT, FOS
42	Qu, M., 2014	[56]	2004 to 2011	China	ND	ND	ND	1652	SF, SB, SS, SD	KB, PCR	NAL, TET, AMP, SXT, GEN, CEP, CFZ, AMC, CTX, CIP, NOR, OFX
43	Rajpara, N., 2018	[57]	2001 to 2010	India	ND	ND	ND	95	SF, SB, SS, SD	KB	AMP, AZI, CTR, CHL, CIP, SXT, CFX, GEN, KAN, NAL, NOR, STR, TET, TMP
44	Sadredinamin, M., 2022	[58]	3/2016 to 9/2018	Iran	ND	2 months to 14 years	185 M, 148 F	333	SF, SS, SB	KB, SAT, PCR	AMP, CTX, CPM, SXT, AZI, CIP, NAL, TET, MIN
45	Sah, S. K., 2019	[59]	4/2016 to 9/2017	Nepal	153	Above 14 years	80 M, 73 F	17	SF, SS	KB	TET, CIP, SXT, CHL, NAL, NOR, AMP, OFX, CTR
46	Salimiyan Rizi, K., 2020	[60]	2/2018 to 9/2019	Iran	233	0–14 years	ND	94	SF, SS, SD	KB, SAT	AZI, CTR, CIP, SXT, NAL, GEN, AMX, AMP, DOX, CFM
47	Shakya, G., 2016	[61]	2003 to 2015	Nepal	ND	All age groups	ND	332	SF, SB, SS, SD	KB	AMP, CIP, NAL, SXT, MEC, CTR
48	Shen, Y., 2013	[62]	1/2008 to 11/2010	China	ND	1–88 years	ND	716	SF, SB, SS, SD	KB, SAT	AMP, AMC, CEP, CTX, GEN, NAL, NOR, TET, SXT
49	Soltan Dallal, M. M., 2018	[63]	5/2015 to 10/2016	Iran	946	Pediatrics	ND	75	SF, SB, SS, SD	KB, DDST, SAT, PCR	GEN, IMI, CHL, NAL, CIP, TET, AMP, SXT, CTX, CAZ, CTR, AZI
50	Tajbakhsh, M., 2012	[64]	9/2008 to 3/2010	Iran	848	2–56 years	ND	37	SF, SB, SS, SD	KB, SAT, PCR	AMC, AMP, TIO, CHL, CIP, GEN, CTX, NAL, TET, TMP
51	Taneja, N., 2021	[65]	2015 to 2019	India	10,456	All age groups	82 M, 55 F	137	SF, SB, SS, SD	KB, SAT	AMP, AMX, CTR, CTX, CIP, SXT, AMK, GEN, CHL, TET
52	Teimourpour, R., 2019	[66]	2015 to 2017	Iran	1280	0–10 years	ND	113	SF, SB, SS, SD	KB, PCR	CIP, NAL, NOR, PER, GEN, CTR, SXT, IMI, AMK, AZI
53	Thompson, C. N., 2015	[67]	5/2009 to 4/2010	Vietnam	1419	0–5 years	908 M, 511 F	48	SF, SS	KB, SAT, DDST	AMP, AMC, CAZ, CIP, GAT, OFX, CHL, TMP, NAL, CTR
54	Ud-Din, A. I., 2013	[4]	2001 to 2011	Bangladesh	ND	All age groups	ND	200	SS	KB, SAT, PCR	AMP, STR, TET, CIP, NAL, MEC, SXT, CTR, CTX, CAZ, IMI
55	Urvashi, 2011	[68]	2004 to 2008	India	12,983	All age groups	ND	106	SF, SB, SS, SD	SF, SB, SS, SD	AMP, SXT, TET, CHL, GEN, NAL, CIP, FUR, CTX, CTR
56	Wang, Y., 2019	[69]	2006 to 2016	China	ND	All age groups	278 M, 196 F	474	SF, SS	E-test, SAT, PCR	CAZ, CTR, CPM, CFP, CFZ, FOX, IMI, NIT, PIP, AMP, TIC, TET, TOB, GEN, AMK, CHL, TCC, LEV, NOR, SXT, AZT
57	Xu, Y., 2016	[70]	1/2001 to 12/2011	China	ND	ND	ND	624	SF	KB, PCR	AMP, AMC, CEP, CTX, GEN, NAL, NOR, TET, SXT
58	Yang, H., 2013a	[71]	2005 to 2011	China	ND	All age groups	163 M, 145 F	308	SF, SB, SS, SD	Agar dilution	AMP, PIP, CTX, CTR, CAZ, CPM, FOX, AZT, NAL, CIP, LEV, NOR, GAT, GEN, AMK, CHL, SXT, TET, IMI
59	Yang, H., 2013b	[72]	2001 to 2008	China	2485	All age groups	287 M, 239 F	526	SF, SS	KB, SAT	CFZ, CTX, AMC, AMP, GEN, TOB, CHL, TET, SXT, NAL, PIM, CIP, FUR
60	Zhang, C. L., 2014	[73]	1/2008 to 12/2012	China	ND	All age groups	ND	356	SF, SS	KB, PCR	AMP, PIP, SAM, TZP, CAZ, CPM, FOX, IMI, SXT, CIP, LEV
61	Zhang, H., 2015	[74]	1/2008 to 12/2013	China	1577	0–14 years	897 M, 680 F	207	SF, SB, SS, SD	KB, SAT	AMP, SAM, CTR, CIP, SXT, CHL
62	Zhang, J., 2014	[75]	1/2004 to 12/2011	China	77,600	All age groups	ND	1638	SF, SS, SB	KB, SAT	TET, TMP, SXT, AMP, AMC, CPM, CTX, CAZ, NAL, CIP, OFX, GEN, STR, CHL
63	Zhang, W. X., 2019	[76]	2010 to 2015	China	ND	ND	ND	402	SF, SS	KB, PCR	AMP, CTX, CPM, STR, CHL, AMC, TET, NAL, CIP, NOR, LEV, FOX, SXT

ND, no data; SF, *Shigella flexneri*; SS, *Shigella sonnei*; SB, *Shigella boydii*; SD, *Shigella dysenteriae*; SAT, slide agglutination test; KB, Kirby–Bauer disk diffusion; DDST, double-disk synergy test; E-test, concentration gradient method; PCR, polymerase chain reaction; AMC, amoxicillin and clavulanic acid; AMK, amikacin; AMP, ampicillin; AMX, amoxicillin; AZI, azithromycin; AZT, aztreonam; CAZ, ceftazidime; CAR, carbenicillin; CPM, cefepime; CEP, cephalothin; CFM, cefixime; CFP, cefoperazone; CFX, cefuroxime; CFZ, cefazolin; CHL, chloramphenicol; CIP, ciprofloxacin; CTR, ceftriaxone; CTX, cefotaxime; DOX, doxycycline; ETP, ertapenem; ERY, erythromycin; SAM, ampicillin and sulbactam; FOS, fosfomycin; FOX, cefoxitin; FUR, furazolidone; GAT, gatifloxacin; GEN, gentamicin; IMI, imipenem; KAN, kanamycin; LEV, levofloxacin; LEX, cefalexin; MEC, mecillinam, MEM, meropenem; MIN, minocycline; NAL, nalidixic acid; NIT, nitrofurantoin; NOR, norfloxacin; OFX, ofloxacin; OXA, oxacillin; PIP, piperacillin; STR, streptomycin; SXT, sulfamethoxazole and trimethoprim; TCC, ticarcillin and clavulanic acid; TET, tetracycline; TIC, ticarcillin; TIO, ceftiofur; TMP, trimethoprim; TOB, tobramycin; TZP, piperacillin and tazobactam. ^1^ Abbreviations of antibiotics are in accordance with the Clinical and Laboratory Standard Institute (CLSI) Guidelines Published in 2020.

**Table 2:** Pooled prevalence of *Shigella* in different Asian regions.

Subgroup	Prevalence (%) [95% CIs ^1^]	No. of Studies	Sample Size (*Shigella* Isolates)	Sample Population	*I* ^2^	*p*-Value
*Regions*						
East Asia	9.5 [0.2–18.7]	4	2389	82,846	100%	<0.01
Middle East	10.2 [4.0–16.3]	12	1409	65,350	98%	<0.01
South Asia	7.4 [4.7–10.2]	16	4690	63,422	100%	0
Southeast Asia	2.9 [2.5–3.3]	4	225	7748	20%	0.29
*Countries*						
Bangladesh	20.3 [19.6–20.9]	1	2925	14,428	NA ^2^	NA
Cambodia	3.0 [2.4–3.7]	2	96	3163	16%	0.27
China	12.1 [1.3–23.0]	3	2371	81,662	100%	<0.01
India	6.2 [3.4–9.0]	10	1598	46,139	99%	<0.01
Iran	10.2 [4.0–16.3]	12	1409	65,350	98%	<0.01
Nepal	7.0 [2.6–11.4]	5	167	2855	93%	<0.01
Taiwan	1.5 [0.9–2.4]	1	18	1184	NA	NA
Vietnam	2.9 [2.1–3.7]	2	129	4585	55%	0.14

^1^ CI, confidence interval; ^2^ NA, not available.

**Table 3:** Pooled prevalence estimates of antimicrobial drug-resistant *Shigella* spp. tested against 49 antibiotics in Asia.

Antibiotics	Prevalence (%) [95% CIs ^1^]	No. of Resistant Isolates	No. of Studies	*I* ^2^	*p*-Value
*First Generation Cephalosporins*					
Cephalothin (CEP)	33.6 [10.7–56.5]	780	7	100%	0
Cefazolin (CFZ)	20.4 [11.8–28.9]	452	5	97%	<0.01
Cefalexin (LEX)	56.4 [0.0–100.0]	40	2	99%	<0.01
*Second Generation Cephalosporins*					
Cefuroxime (CFX)	10.9 [2.0–19.7]	44	4	94%	<0.01
Cefoxitin (FOX)	1.8 [0.4–3.1]	36	7	78%	<0.01
*Third Generation Cephalosporins*					
Ceftazidime (CAZ)	20.3 [8.8–31.7]	464	23	100%	0
Cefixime (CFM)	27.6 [7.6–47.7]	199	8	99%	<0.01
Cefoperazone (CFP)	20.0 [9.8–30.2]	162	3	79%	<0.01
Ceftriaxone (CTR)	23.8 [16.1–31.6]	1035	39	98%	0
Cefotaxime (CTX)	28.6 [19.7–37.5]	1938	34	99%	0
Ceftiofur (TIO)	21.6 [9.8–38.2]	8	1	NA ^2^	NA
*Fourth Generation Cephalosporins*					
Cefepime (CPM)	19.2 [6.4–32.1]	645	14	99%	<0.01
*Aminoglycosides*					
Amikacin (AMK)	15.9 [6.9–24.8]	234	20	95%	<0.01
Gentamicin (GEN)	21.7 [14.7–28.6]	2494	36	99%	0
Kanamycin (KAN)	51.9 [0.8–100.0]	91	2	98%	<0.01
Streptomycin (STR)	98.4 [96.9–100.0]	2279	6	78%	<0.01
Tobramycin (TOB)	4.7 [0.7–8.6]	91	4	91%	<0.01
*Carbapenems*					
Ertapenem (ETP)	0.0 [0.0–1.1]	0	3	0%	1.00
Imipenem (IMI)	0.1 [0.0–0.2]	45	21	80%	<0.01
Meropenem (MEM)	0.0 [0.0–0.7]	0	5	0%	1.00
*Macrolides*					
Azithromycin (AZI)	29.2 [20.8–37.6]	389	17	95%	<0.01
Erythromycin (ERY)	57.4 [51.6–63.2]	160	2	0%	0.73
*Monobactams*					
Aztreonam (AZT)	15.0 [5.6–24.5]	172	6	94%	<0.01
*Penicillins*					
Amoxicillin + clavulanic acid (AMC)	23.4 [12.4–34.4]	1687	17	99%	0
Amoxicillin (AMX)	73.2 [52.4–94.1]	240	5	98%	<0.01
Ampicillin (AMP)	72.6 [66.4–78.7]	12,667	57	99%	0
Ampicillin + sulbactam (SAM)	27.5 [15.1–39.9]	307	9	97%	<0.01
Carbenicillin (CAR)	49.9 [15.4–84.5]	87	2	95%	<0.01
Mecillinam (MEC)	37.8 [8.8–66.7]	191	3	98%	<0.01
Oxacillin (OXA)	71.9 [53.3–86.3]	23	1	NA	NA
Piperacillin (PIP)	43.5 [22.8–64.2]	740	7	99%	<0.01
Piperacillin + tazobactam (TZP)	0.2 [0.0–0.6]	1	5	0%	0.98
Ticarcillin (TIC)	90.5 [84.7–96.3]	884	3	89%	<0.01
Ticarcillin + clavulanic acid (TCC)	11.7 [0.0–27.1]	55	3	94%	<0.01
*Phenicols*					
Chloramphenicol (CHL)	36.3 [27.0–45.6]	2905	32	100%	0
*Quinolones*					
Ciprofloxacin (CIP)	29.8 [22.4–37.1]	2476	54	99%	0
Gatifloxacin (GAT)	4.6 [0.0–13.9]	29	2	95%	<0.01
Levofloxacin (LEV)	14.7 [6.1–23.3]	212	12	92%	<0.01
Nalidixic acid (NAL)	68.0 [58.6–77.4]	9656	44	100%	0
Norfloxacin (NOR)	24.4 [14.4–34.4]	1287	20	99%	0
Ofloxacin (OFX)	31.3 [16.9–45.7]	839	18	99%	0
*Tetracyclines*					
Doxycycline (DOX)	48.0 [7.0–89.0]	154	3	99%	<0.01
Minocycline (MIN)	57.1 [23.5–90.6]	202	4	98%	<0.01
Tetracycline (TET)	78.3 [70.4–86.3]	7037	35	99%	0
*Others*					
Fosfomycin (FOS)	1.4 [0.4–3.2]	5	1	NA	NA
Furazolidone (FUR)	9.1 [0.0–23.9]	21	3	92%	<0.01
Nitrofurantoin (NIT)	0.0 [0.0–0.2]	0	2	0%	1.00
Sulfamethoxazole + trimethoprim (SXT)	78.6 [74.4–82.7]	13,991	56	98%	0
Trimethoprim (TMP)	95.5 [92.3–98.8]	1780	4	55%	0.08

^1^ CI, confidence interval; ^2^ NA, not available.

**Table 4:** Pooled prevalence of multidrug-resistant *Shigella* in different Asian regions.

Subgroup	Prevalence (%) [95% CIs ^1^]	No. of MDR ^2^ Isolates	Total *Shigella* Isolates	No. of Studies	*I* ^2^	*p*-Value	
*Regions*							
East Asia	73.2 [58.5–87.9]	4756	5755	13	100%	0	
Middle East	54.3 [38.1–70.4]	537	1107	13	99%	<0.01	
South Asia	77.7 [66.1–89.3]	1348	1794	10	98%	<0.01	
Southeast Asia	83.4 [53.8–100.0]	109	136	2	97%	<0.01	
*Countries*							
Bangladesh	94.0 [89.8–96.9]	188	200	1	NA ^3^	NA	
Cambodia	98.2 [90.3–100.0]	54	55	1	NA	NA	
China	76.3 [61.8–90.8]	4733	5689	12	100%	0	
India	79.6 [66.8–92.4]	955	1197	7	97%	<0.01	
Iran	58.4 [38.7–78.1]	410	731	11	99%	<0.01	
Kuwait *	50.0 [34.2–65.8]	21	42	1	NA	NA	
Nepal	62.8 [29.6–96.0]	205	397	2	97%	<0.01	
South Korea	34.8 [23.5–47.6]	23	66	1	NA	NA	
Turkey	28.6 [22.9–34.9]	67	234	1	NA	NA	
U.A.E *	39.0 [29.4–49.3]	39	100	1	NA	NA	
Vietnam	67.9 [56.6–77.8]	55	81	1	NA	NA	

* Data from Kuwait and the U.A.E were reported by a single study (Jamal, W., 2010) [33]. ^1^ CI, confidence interval; ^2^ MDR, multidrug-resistant; ^3^ NA, not available.

**Table 5:** Pooled prevalence of ESBL-producing *Shigella* spp. in different Asian regions.

Subgroup	Prevalence (%) [95% CIs ^1^]	No. of ESBL ^2^ Isolates	Total *Shigella* Isolates	No. of Studies	*I* ^2^	*p*-Value	
*Regions*							
East Asia	31.0 [0.9–61.0]	218	870	3	99%	<0.01	
Middle East	20.7 [6.7–34.7]	84	427	7	93%	<0.01	
South Asia	9.7 [2.3–17.1]	31	248	4	79%	<0.01	
Southeast Asia	52.5 [6.8–98.3]	63	117	2	97%	<0.01	
*Countries*							
Cambodia	29.1 [17.6–42.9]	16	55	1	NA ^3^	NA	
China	31.0 [0.9–61.0]	218	870	3	99%	<0.01	
India	10.5 [1.2–19.8]	30	233	3	86%	<0.01	
Iran	26.3 [8.3–44.3]	75	284	5	93%	<0.01	
Nepal	6.7 [0.2–31.9]	1	15	1	NA	NA	
Turkey	11.6 [3.9–25.1]	5	43	1	NA	NA	
U.A.E	4.0 [1.1–9.9]	4	100	1	NA	NA	
Vietnam	75.8 [63.3–85.8]	47	62	1	NA	NA	

^1^ CI, confidence interval; ^2^ ESBL, extended-spectrum beta-lactamase; ^3^ NA, not available.

## Data Availability

All data relevant to this review are included in the text and references.
